# Prognostic Impact of Pedicle Clamping during Liver Resection for Colorectal Metastases

**DOI:** 10.3390/cancers13010072

**Published:** 2020-12-29

**Authors:** Tobias S. Schiergens, Moritz Drefs, Maximilian Dörsch, Florian Kühn, Markus Albertsmeier, Hanno Niess, Markus B. Schoenberg, Matthias Assenmacher, Helmut Küchenhoff, Wolfgang E. Thasler, Markus O. Guba, Martin K. Angele, Markus Rentsch, Jens Werner, Joachim Andrassy

**Affiliations:** 1 Department of General, Visceral and Transplant Surgery, Ludwig-Maximilians-University Munich, Marchioninistr 15, D-81377 Munich, Germany; moritz.drefs@med.lmu.de (M.D.); doerschmaximilian@gmail.com (M.D.); florian.kuehn@med.lmu.de (F.K.); malberts@med.lmu.de (M.A.); hanno.niess@med.lmu.de (H.N.); markus.schoenberg@med.lmu.de (M.B.S.); wolfgang.thasler@swmbrk.de (W.E.T.); markus.guba@med.lmu.de (M.O.G.); martin.angele@med.lmu.de (M.K.A.); markus.rentsch@klinikum-ingolstadt.de (M.R.); jens.werner@med.lmu.de (J.W.); joachim.andrassy@med.lmu.de (J.A.); 2 Department of Statistics, Ludwig-Maximilians-University Munich, Akademiestr 1, D-80799 Munich, Germany; matthias.assenmacher@stat.uni-muenchen.de (M.A.); kuechenhoff@stat.uni-muenchen.de (H.K.)

**Keywords:** colorectal liver metastasis, pedicle clamping, Pringle, transfusion, blood loss

## Abstract

**Simple Summary:**

During liver resection for colorectal cancer metastases, the flow of blood into the liver can be technically interrupted, which is also referred to as pedicle clamping or the Pringle maneuver. The effect on long-term oncologic outcomes is still under debate with respect to mechanisms of ischemia-reperfusion as well as transfusion demand and earlier disease recurrence. In this retrospective cohort study, the effect of pedicle clamping on the overall and disease-free survival of 336 patients undergoing curative resection for colorectal cancer liver metastases was analyzed with univariate, multivariate, and propensity-score methods. Favorable long-term outcomes and lower rates of increased transfusion demand were observed in patients with pedicle clamping while no increased postoperative morbidity was monitored. Further prospective evaluation of potential oncologic benefits of pedicle clamping in these patients may be meaningful.

**Abstract:**

Pedicle clamping (PC) during liver resection for colorectal metastases (CRLM) is used to reduce blood loss and allogeneic blood transfusion (ABT). The effect on long-term oncologic outcomes is still under debate. A retrospective analysis of the impact of PC on ABT-demand regarding overall (OS) and recurrence-free survival (RFS) in 336 patients undergoing curative resection for CRLM was carried out. Survival analysis was performed by both univariate and multivariate methods and propensity-score (PS) matching. PC was employed in 75 patients (22%). No increased postoperative morbidity was monitored. While the overall ABT-rate was comparable (35% vs. 37%, *p* = 0.786), a reduced demand for more than two ABT-units was observed (*p* = 0.046). PC-patients had better median OS (78 vs. 47 months, *p* = 0.005) and RFS (36 vs. 23 months, *p* = 0.006). Multivariate analysis revealed PC as an independent prognostic factor for OS (HR = 0.60; *p* = 0.009) and RFS (HR = 0.67; *p* = 0.017). For PC-patients, 1:2 PS-matching (*N* = 174) showed no differences in the overall ABT-rate compared to no-PC-patients (35% vs. 40%, *p* = 0.619), but a trend towards reduced transfusion requirement (>2 ABT-units: 9% vs. 21%, *p* = 0.052; >4 ABT-units: 2% vs. 11%, *p* = 0.037) and better survival (OS: 78 vs. 44 months, *p* = 0.088; RFS: 36 vs. 24 months; *p* = 0.029). Favorable long-term outcomes and lower rates of increased transfusion demand were observed in patients with PC undergoing resection for CRLM. Further prospective evaluation of potential oncologic benefits of PC in these patients may be meaningful.

## 1. Introduction

The liver represents the most frequent site of distant metastases of colorectal cancer [1]. Liver resection for colorectal liver metastases (CRLM) with multimodality treatment strategies is considered safe and oncologically effective, improving survival and quality of life [2,3,4]. Multidisciplinary therapy has markedly evolved since effective conversion therapy as well as new surgical treatment strategies have led to increased resectability with more and more aggressive surgical approaches being justified [4,5,6]. Extended liver resections bear a relevant risk of severe hemorrhage as a large amount of parenchyma has to be sacrificed [4]. Although the causality remains unclear, perioperative blood loss and the associated need for allogeneic blood transfusion (ABT) appear not only to be associated with adverse perioperative events but also with oncologic outcomes such as tumor recurrence and survival [7,8,9,10]. Cancer recurrence due to transfusion may be ascribed to perioperative transfusion-related immune modulation [10,11]. Of note, approximately one-third of patients require perioperative blood transfusion during liver resection depending on the study population and the transfusion trigger standards [7,12,13]. These data emphasize the need for reasonable transfusion triggers and blood saving techniques [14]. Among the latter, pedicle clamping (PC), also referred to as the Pringle maneuver [15], results in decreased hepatic inflow and is routinely applied. However, PC is still under debate regarding its tolerable duration and potential negative oncologic effects due to hepatic ischemia-reperfusion. Most studies in the literature found no significant negative impact of PC on cancer recurrence in CRLM patients [16]. In a large cohort of patients undergoing hepatic resection for both benign and malignant lesions at our center, PC was not associated with increased morbidity or poorer overall survival [8]. Reducing blood loss and ABT requirement by adequate application of PC in CRLM-patients might therefore represent a feasible approach not only to improve short-term but also long-term outcomes.

The aim of this study was to analyze the effect of PC on perioperative ABT as well as recurrence-free (RFS) and overall survival (OS) in patients undergoing liver resection for CRLM.

## 2. Results

### 2.1. Patients’ and Tumor Characteristics

A total of 336 patients were included in the present study with a median follow-up of 37 months. Of them, 75 patients (22%) underwent PC during liver resection with a median clamping duration of 12 min (5–80) per patient. In the majority of PC-patients, it was employed for less than 20 min overall (*N* = 52, 69%). Only 11% of the PC-group underwent the maneuver for more than 30 min in total. Patients’ characteristics and oncologic data are summarized in Table 1, including statistical comparison of PC-patients and no-PC-patients.

Comparison of PC and no-PC-patients revealed the proportion of male patients and those with anatomic resections to be higher in the PC-group (Table 1). No other significant differences were observed. Of note, there were no differences in potential confounders such as co-morbidities (American Society of Anesthesiologists (ASA) grade, Charlson co-morbidity index), primary tumor stage, preoperative serum tumor markers, number of liver lesions, postoperative morbidity including post-hepatectomy liver failure (PHLF), resection status (free resection margins) as well as additive chemotherapy. In the PC-group, however, more patients tended to have metachronous disease (73% vs. 61%) and larger tumors (largest diameter ≥ 50 mm, 27% vs. 16%).

### 2.2. Pedicle Clamping, Blood Loss and Transfusion

Overall, no difference in estimated intraoperative blood loss (EBL) was observed between PC- and no-PC-patients (Table 1). Upon stratification into major and minor liver resections or anatomic and non-anatomic (atypical) resections, also no differences in EBL were observed, respectively. The number of patients with major EBL, however, tended to be higher in the PC-group (32% vs. 23%). When comparing the number of patients receiving perioperative ABT and the number of ABT-units transfused, no relevant differences were observed between PC-patients and those without clamping. However, despite a trend towards a higher blood loss, increased transfusion demand was observed to be significantly lower in PC-patients (8% vs. 18%; *p* = 0.046).

### 2.3. Survival Analysis

**Figure 1:**
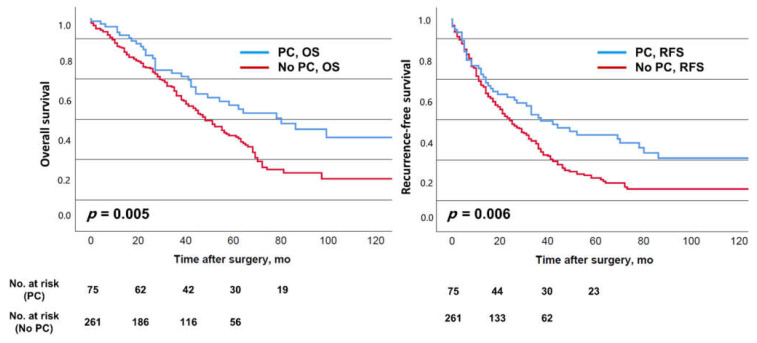
Overall survival (OS) and recurrence-free survival (RFS) of the entire cohort depending on the employment of pedicle clamping (PC).

Median OS and RFS for the entire study population were 51 and 26 months, respectively. The corresponding 5-year survival rates were 57% and 42%. Patients with PC were observed to have better median OS (78 vs. 47 months, *p* = 0.005) and RFS (36 vs. 23 months, *p* = 0.006) compared to patients without PC, with 5-year survival rates of 57% vs. 43% (OS) as well as 42% vs. 21% (RFS), respectively (Figure 1).

Duration of the PC maneuver (min) was not significantly associated with OS (*p* = 0.356, Hazard Ratio 1.01) or RFS (*p* = 0.309, Hazard Ratio 1.01) upon Cox regression analysis. Univariate survival analysis for both OS and RFS revealed patient age (OS: *p* < 0.001; RFS: *p* = 0.029), the Charlson co-morbidity index (OS and RFS *p* < 0.001, respectively), the presence of more than three metastases (OS and RFS *p* < 0.001, respectively), the need for major hepatic resection (OS: *p* = 0.019; RFS: *p* = 0.016), perioperative ABT (OS and RFS *p* < 0.001, respectively) and positive resection margins (R1; OS: *p* = 0.001; RFS: *p* < 0.001) as significant risk factors. Major complications were able to significantly predict OS (*p* = 0.024). Of note, EBL or major EBL was not significantly associated with OS or RFS. In the final multivariable Cox-models (Table 2), PC, the presence of more than three metastases, positive resection margins, co-morbidities, and transfusion were independent prognostic factors for both OS and RFS.

In addition, age was an independent risk factor for shortened OS. Of note, variables that were associated with clamping such as gender or the type of resection as well as those factors showing a statistical trend (such as timing of metastasis development or maximum diameter) were not significantly associated with OS or RFS.

### 2.4. Propensity-Score Matching

The data of 174 patients were entered into a final 1:2 propensity-score (PS)-matching model as this showed the best balance in covariates between PC- and no-PC-patients. It was assessed evaluating the standardized mean differences, which were below 0.10 except for one variable. The results of PS-matching and the comparison of PS-PC-patients to PS-no-PC-patients regarding the matching variables are shown in Table 3.

**Figure 2:**
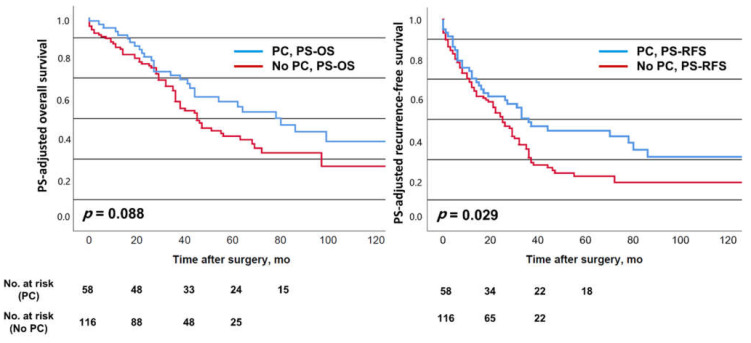
Propensity-score (PS) adjusted overall survival (PS-OS) and recurrence-free survival (PS-RFS) depending on the employment of pedicle clamping (PC).

No significant differences were observed for EBL, although there was a trend of less EBL in the PC-group (median of 500 mL vs. 800 mL in the no-PC-group, *p* = 0.206). While the overall transfusion rate was comparable between PS-PC-patients and PS-no-PC-patients (35% vs. 40%, *p* = 0.619), the number of those receiving more than four units of blood was significantly lower in the PS-PC-group (>2 units: 9% vs. 21%; *p* = 0.052; >4 units: 2% vs. 11%, *p* = 0.037). Results of the PS-adjusted survival analysis are shown in Figure 2.

Median OS tended to be better in the PS-PC-group (78 vs. 44 months, *p* = 0.088) and RFS was observed to be significantly improved compared to the PS-no-PC-group (36 vs. 24 months, *p* = 0.029). Five-year survival rates were 58% (PS-PC) vs. 41% (PS-no-PC) for OS, and 44% (PS-PC) vs. 22% (PS-no-PC) for RFS, respectively.

## 3. Discussion

The purpose of this study was to evaluate the effect of pedicle clamping on perioperative transfusion and long-term oncologic outcomes in patients undergoing curative resection of CRLM. Patients with PC did not have increased morbidity and mortality. The Pringle maneuver was not associated with lower blood loss or overall transfusion rates, but a reduced demand for a higher number of ABT-units. PC represented an independent factor for better survival in both OS and RFS when adjusted to significant confounding covariables. Propensity-score adjusted survival analysis corroborated these results, also showing a trend towards better long-term oncologic survival in the PC-group.

Favorable outcomes following resection of CRLM with excellent long-term survival rates justify an increasingly aggressive surgical approach in these patients [4]. A multitude of risk factors associated with worse survival has been reported, and these were entered into different prognostic scores with the aim of risk stratification and patient selection [17,18,19]. Blood loss and the consecutive need for perioperative transfusion during liver resection for CRLM still represent a relevant problem as they bear the risk of unfavorable short- and long-term outcomes [13,20,21]. Intraoperative blood loss is thereby associated with biological characteristics of the tumor and the extent of surgery [22]. Regarding long-term oncologic survival, the association of allogeneic blood transfusion and earlier recurrence in both primary and metastatic CRC has been traced to an immunologic phenomenon called transfusion-related immune modulation (TRIM) with clinical as well as laboratory and animal experiment-based evidence that stored blood or erythrocytes may have cancer-promoting effects [11]. In addition, the “freshness” of blood products has been shown to be of relevance for the postoperative outcome [20]. The association of transfusion and recurrence, however, has also been critically discussed in the literature with results showing no adverse long-time effects [23,24]. Causal relationships have not been convincingly expounded and risk factors for developing disease recurrence are multifactorial depending on both clinical factors [5,18,19,25] and tumor biology [4,26,27,28,29].

In this context, however, attempts to reduce blood loss and avoid transfusion have become of great interest as potential approaches to improve short- and long-term outcomes. To diminish blood loss, PC has been established in hepatobiliary surgery. Its effectiveness in reducing blood loss and transfusion requirements has been shown [30,31], however, other studies revealed comparable blood loss between the PC and no-PC-group [32,33]. A meta-analysis showed no beneficial effect on perioperative outcome [34]. In the present study, no difference in EBL was observed between PC- and no-PC-patients; the number of patients with major EBL tended to be even higher in the PC-group. As PC was employed on demand at the individual discretion of the surgeon at resections with impending major blood loss, the PC-group might therefore represent a high-risk group. After PS-matching, a trend of less EBL in the PC-group was seen, but this was not statistically significant. In addition, we did not observe lower overall transfusion rates in the PC-group, but a reduced demand for a higher number of ABT-units.

A survey among European surgeons on the indications and techniques of PC published by van der Bilt et al. [35] revealed that PC is never applied by 10% of the respondents on indication by 71% of them, and routinely by 19% of them. Routine clamping was observed to be particularly performed by high-volume and senior surgeons [35]. In addition, there is an ongoing discussion about the relevance of the duration of PC. No upper time limit was reported and even applications for more than one hour were observed not to result in increased mortality or morbidity, i.e., postoperative liver failure [36,37,38]. However, hepatic co-morbidities such as steatohepatitis were reported to be a problem in patients undergoing prolonged PC, resulting in a higher risk of septic complications [39]. In contrast to Weiss et al. (15%), PC was performed for more than 60 min in only three patients in our study. Overall, the employment as well as the duration of PC in the present cohort can be considered moderate.

Studies evaluating the long-term effects of PC in patients undergoing liver resection for CRLM are rare. In a meta-analysis published in 2013, Matsuda et al. [16] included four studies [38,40,41,42]. The authors came to the conclusion that PC was not associated with oncologic outcomes [16]. Weiss et al. reported one of the aforementioned studies with no association between PC and RFS or OS [38]. In their study (94%) and in that of Giuliante et al. (65%) [41], the employment of PC was far more often than in our cohort (22%). Tsang et al. reported a PC-rate of 27% among CRLM patients [43] whereas it was balanced in the cohorts published by Wong et al. [42] and Ferrero et al. [40]. Two studies reported PC to be associated with the number of hepatic lesions [38,41]. Weiss et al. also found the type of resection to be different in the PC-group compared to the group without PC [38]. In the publications of Ferrero and Wong, no significant differences were found between these groups when looking at pre- and perioperative characteristics. In the present analysis, PC was more often employed in male patients and in those with anatomic resections, whereas there were no relevant differences in prognostic variables. In another study, Olthof et al. reported results of 208 CRLM patients, of whom 64 (31%) underwent PC [44]. Of those PC-patients, 40 were observed having severe ischemia, defined as ≥20 min continuous or ≥45 min cumulative intermittent PC. This subgroup of severe ischemia was shown to have worse RFS and OS, indicating that prolonged PC may result in worse oncologic long-term outcomes.

Experimental studies on the effect of hepatic ischemia-reperfusion on the outgrowth of cancer cells revealed hepatocyte dysfunction and increased inflammatory cytokines such as TNF-α as well as matrix metalloproteinases [45,46,47,48,49]. This may occur during PC and bear the risk of worse oncologic outcomes. Furthermore, remnant liver ischemia after resection of CRLM leaving devitalized liver tissue behind has also been reported to be linked to worse cancer-specific survival [50]. However, the duration and severity of ischemia-reperfusion has to be taken into account. In the present study, PC was applied for a median of only 12 min and can be considered mild to moderate. The observations of this study suggest that PC, when moderately applied, may result in favorable oncologic outcomes, perhaps by reducing higher demands of allogeneic blood transfusion. A reduction of 20% in OS is considered clinically meaningful in this patient group [38]; this was the case for the present OS analysis (univariate: 78 vs. 47 months; PS-analysis: 78 vs. 44 months; multivariable analysis: HR = 0.60). For RFS, these results were less distinct (univariate: 36 vs. 23 months; PS-analysis: 36 vs. 24 months; multivariable analysis: HR = 0.67), whereas we could observe a favorable long-term effect in the Kaplan–Meier plots of RFS (Figure 1 and Figure 2) as they widened later in the postoperative course. This was underlined by relevant differences in the 5-year survival rates. Interestingly, the meta-analysis of the effect of PC on recurrence and survival, including a total of 2114 patients (Matsuda et al. [16]), showed a non-significant favorable trend for PC for intrahepatic recurrence (odds ratio (OR) 0.88 (0.69–1.11)) and RFS (OR 0.88 (0.70–1.10)), but not OS (OR 0.99 (0.79–1.24)). Even though this favorable oncologic effect observed in the present study is worth being very critically discussed, our data emphasize that moderate PC does not seem to adversely affect short-term and long-term survival after resection of CRLM.

This study has several limitations and its results must be critically discussed. First, it is significantly limited by its retrospective nature and associated biases, i.e., selection bias. Second, it represents a single-center experience with a limited patient number and the employment of PC may not only vary between surgeons but also different hepatobiliary centers. Third, the inherent key issue is the question whether the association between PC, ABT, and the outcome variables analyzed represents a causative effect or if there were unmanageable confounders acting inwardly. It must be critically discussed that this intraoperative maneuver of several minutes has such a tremendous impact on long-term survival. Fourth, we did not observe differences in EBL or overall transfusion rates between PC- and no-PC-patients, but only differences in the requirement for higher numbers of ABT-units. Fourth, the propensity-score analysis resulted in a smaller sample size with loss of observations and statistical power making a stronger conclusion difficult.

As PC represents an individual maneuver in a complex surgical situation, the results of studies focusing on PC have to be interpreted in the context of their design and surgical era [51]. In this context, the lack of influence of PC on EBL might therefore not be surprising. In addition, new endpoints will be of importance in future studies such as intestinal congestion and bowel movement recovery [51].

## 4. Patients and Methods

### 4.1. Design and Study Population

From a prospectively maintained database, demographic, clinical, laboratory, and perioperative data, including application and duration of PC as well as RFS and OS of patients undergoing elective, curative-intended liver resection for CRLM between 2003 and 2014 at our institution were retrospectively analyzed. Data were collected using standardized electronic case report forms and stratified into clinical scores where applicable as previously described in detail [7]. PC was not routinely conducted but performed when deemed indicated at the surgeon’s discretion in cases of impending or increasing blood loss. As standard, PC was performed for a maximum length of 20 min in continuity followed by 5–10 min of unclamping. Parenchymal division during hepatic resection was performed using a cavitron ultrasonic surgical aspirator (CUSA) and/or the clamp–crush technique for small atypical (wedge) resections. Major EBL was defined as intraoperative EBL of more than 1000 mL. Increased transfusion demand was defined as the requirement of more than two perioperative ABT-units. The study was approved by the Ethics Committee, Faculty of Medicine, Ludwig-Maximilians-University (LMU), Munich, Germany. Design, data acquisition, statistical methods, and manuscript preparation were carried out according to STROBE guidelines for strengthening of reporting of observational studies [52].

### 4.2. Statistical Analysis

Results were expressed as median and range (minimum and maximum) or as mean values ± standard deviation (SD). For comparison of variables, Χ^2^-test or Fisher’s exact test (cases of low frequency) were used where appropriate depending on the variable. For comparison of continuous variables, the Mann–Whitney U test for non-parametric analysis was applied. Univariate survival analysis (RFS, OS) was performed by estimating Kaplan–Meier curves and applying the log-rank test for statistical discrimination and by Cox regression for continuous variables. The number of patients at risk per group illustrated by the Kaplan–Meier survival estimator was truncated when it was less than one-third of the patient number at surgery. For multivariable modeling of RFS and OS, Cox’s proportional hazard model applying a forward inclusion procedure was calculated for factors featuring significant univariate association and those being hypothesized for adjustment. In case of multivariable analysis of survival, the Hazard Ratio (HR) with its 95% confidence interval (95% CI) was given. In addition, PS-matching was performed. It was conducted for empirically and potential prognostic confounders as well as variables that were shown to be relevantly different when PC-patients and no-PC-patients were compared in order to improve structural equality of groups. These weighted factors included gender, patient age at resection of CRLM, the stage of the primary tumor (T-stage or N-stage, respectively), synchronous vs. metachronous occurrence of hepatic metastasis, elevated serum carcinoembryonic antigen (CEA), type of resection (anatomic vs. non-anatomic) and the number as well as the largest diameter of liver lesions. A 1:2 PS-matching (PC vs. no-PC) showed the best balance in covariates between PC- and no-PC-patients. It was assessed evaluating the standardized mean differences which were below 0.10 except for one variable.

In general, *p* < 0.05 was regarded as statistically significant. For statistical analysis, SPSS statistical software package (version 25, IBM, Chicago, IL, USA) and R [53] (packages R-Studio [54], foreign, ggplot2 [55] survival, mgcv, Matching, and survminer) were used.

## 5. Conclusions

In patients undergoing liver resection for colorectal liver metastases, intraoperative moderate pedicle clamping was observed to be associated with favorable long-term outcomes. Although we did not observe differences in blood loss or overall transfusion rates between PC- and no-PC-patients, a lower demand for higher numbers of ABT-units was seen in PC-patients. In addition, no adverse short-outcomes were monitored. Thus, pedicle clamping can be safely employed in these patients. Our data require further prospective evaluation of the potential oncologic benefit of pedicle clamping in a randomized trial.

## Figures and Tables

**Table 1:** Clinical characteristics of patients with and without pedicle clamping (PC).

Characteristic	All Patients *N* (%)	No PC *N* (%)	PC *N* (%)	*p* ^1^
No. of patients	336 (100)	261 (77.7)	75 (22.3)	
Median Age (range)	62 (21–86)	64 (21–86)	63 (41–82)	0.610
Gender Female Male	114 (34) 222 (66)	98 (38) 163 (62)	16 (21) 59 (79)	0.009
ASA > 2	226 (67)	174 (67)	52 (69)	0.388
Charlson comorbidity index (CCI)	8 (8–13)	8 (8–13)	8 (8–10)	0.737
Hepatic pre-conditions Steatosis Cirrhosis	185 (55) 3 (1)	138 (53) 3 (1)	47 (63) 0	0.148 0.100
Primary tumor Colon Rectum	174 (52) 162 (48)	136 (52) 125 (48)	38 (51) 37 (49)	0.896
Primary tumor stagepT3/pT4 pN+	240 (71) 179 (53)	181 (69) 140 (54)	59 (79) 39 (52)	0.845 0.184
Neoadjuvant chemotherapy	241 (72)	186 (71)	55 (73)	0.773
Tumor markers CEA elevated CA 19-9 elevated	130 (39) 84 (25)	102 (39) 66 (25)	28 (37) 18 (24)	0.685 0.650
Timing of development Synchronous ^2^ Metachronous	122 (36) 214 (64)	102 (39) 159 (61)	20 (27) 55 (73)	0.057
>3 hepatic metastases	47 (14)	37 (14)	10 (13)	1.000
Maximum metastasis diameter ≥ 50 mm	63 (19)	43 (16)	20 (27)	0.064
Extent of liver resection Major hepatectomy ^3^ Minor hepatectomy	116 (35) 220 (65)	90 (34) 171 (66)	26 (35) 49 (65)	0.100
Type of resection Anatomic Non-anatomic (wedge/atypical)	235 (70) 101 (30)	173 (66) 88 (34)	62 (83) 13 (17)	0.006
Duration of resection (min)	180 (60–580)	180 (60–580)	180 (70–310)	0.559
Estimated blood loss (mL)	650 (100–6000)	700 (100–6000)	600 (100–6000)	0.502
Major estimated blood loss ^4^	83 (25)	59 (23)	24 (32)	0.100
Perioperative ABT ^5^	123 (37)	97 (37)	26 (35)	0.786
Increased ABT-demand ^5,6^	52 (15)	46 (18)	6 (8)	0.046
Number of perioperative ABT ^5^	1.48 ± 3.80	1.66 ± 4.20	0.84 ± 1.80	0.393
Major complications	80 (24)	59 (23)	21 (28)	0.361
Post hepatectomy liver failure	15 (4)	13 (5)	2 (3)	0.536
ICU	148 (44)	114 (44)	34 (45)	0.790
ICU length of stay	2.3 ± 6.9	2.4 ± 7.3	2.0 ± 5.3	0.595
30-day-mortality	7 (2)	6 (2)	1 (1)	0.513
90-day-mortality	14 (5)	13 (5)	1 (1)	0.207
Resection margins involved (R1)	25 (7)	18 (7)	7 (9)	0.461
Additive chemotherapy ^7^ (*N* = 282)	159/282 (56)	122/214 (57)	37/68 (54)	0.779

^1^ Comparison between PC and no PC; ^2^ Assessed before or within three months after primary tumor resection; ^3^ Defined as resection of more than three liver segments; ^4^ defined as intraoperative blood loss of more than 1000 mL; ^5^ ABT: allogeneic blood transfusion; ^6^ defined as perioperative transfusion of more than two ABT-units; ^7^ Lost to follow-up for additive chemotherapy: all patients 16%; no PC: 18%; PC: 9%.

**Table 2:** Multivariable analysis of recurrence-free and overall survival.

Prognostic Factor	Recurrence-Free Survival		
	*p*	HR ^1^	95%-CI ^2^
Pedicle clamping (Pringle)	0.017	0.67	0.48–0.93
>3 hepatic metastases	0.011	1.63	1.12–2.36
Allogeneic blood transfusion	0.002	1.05 ^3^	1.02–1.08
Charlson co-morbidity index	0.014	1.23	1.04–1.45
Positive resection margins	0.003	2.08	1.29–3.34
	**Overall Survival**		
	***p***	**HR ^1^**	**95%-CI ^2^**
Age > 70 years	0.034	1.43	1.03–1.98
Pedicle clamping (Pringle)	0.009	0.60	0.41–0.88
>3 hepatic metastases	0.012	1.68	1.12–2.51
Allogeneic blood transfusion	0.001	1.06 ^3^	1.02–1.10
Charlson co-morbidity index	0.009	1.30	1.07–1.57
Positive resection margins	<0.001	3.18	1.92–5.27

^1^ HR: Hazard Ratio; ^2^ 95%-CI: 95% confidence-interval; ^3^ HR for the number of ABT-units.

**Table 3:** Results of propensity-score matching.

Matching Variable	SMD ^1^ Before PS-Matching	SMD ^1^ After PS-Matching	*p ^2^*
Gender	0.26	0.08	0.722
Age	0.01	0.05	0.707
Primary tumor stage (pT stage)	0.14	0.11	0.628
Primary tumor stage (pN stage)	0.20	0.09	0.608
Synchronous vs. metachronous occurrence of CRLM	0.22	0.09	0.600
Serum CEA elevated	0.12	0.07	0.869
Type of CRLM resection (anatomic vs. non-anatomic)	0.50	0.07	0.662
Number of liver lesions	0.06	0.02	0.857
Largest diameter of liver lesions	0.08	0.04	0.987

^1^ Standardized mean differences; ^2^ Comparison of variables between PC and no PC.

## Data Availability

The datasets used and/or analyzed during the current study are available from the corresponding author on reasonable request.
